# Phylogenetic Analysis of Bird-Virulent West Nile Virus Strain, Greece

**DOI:** 10.3201/eid2512.181225

**Published:** 2019-12

**Authors:** George Valiakos, Konstantinos Plavos, Alexandros Vontas, Marina Sofia, Alexios Giannakopoulos, Themis Giannoulis, Vassiliki Spyrou, Constantina N. Tsokana, Dimitrios Chatzopoulos, Maria Kantere, Vasilis Diamantopoulos, Angeliki Theodorou, Spyridoula Mpellou, Athanasios Tsakris, Zissis Mamuris, Charalambos Billinis

**Affiliations:** University of Thessaly, Karditsa, Greece (G. Valiakos, K. Plavos, A. Vontas, M. Sofia, A. Giannakopoulos, T. Giannoulis, V. Spyrou, C.N. Tsokana, D. Chatzopoulos, M. Kantere, Z. Mamuris, C. Billinis);; Public Health Director, Region of Peloponnese, Tripoli, Greece (V. Diamantopoulos);; Directorate-General for Regional Agricultural Economics and Veterinary, Region of Peloponnese, Nafplio, Greece (A. Theodorou);; Bioefarmoges Eleftheriou LP—Integrated Mosquito Control, Marathon, Greece (S. Mpellou);; University of Athens, Athens, Greece (A. Tsakris)

**Keywords:** emerging or reemerging diseases, genome sequence, surveillance, West Nile virus, wild birds, zoonoses, viruses, Greece

## Abstract

We report the full polyprotein genomic sequence of a West Nile virus strain isolated from Eurasian magpies dying with neurologic signs in Greece. Our findings demonstrate the local genetic evolution of the West Nile virus strain responsible for a human disease outbreak in the country that began in 2010.

West Nile virus (WNV) is the etiologic agent of an ongoing human disease outbreak in Greece since 2010. Until 2014, successive yearly outbreaks occurred mainly in central and northeastern Greece (*1*). After a 2-year hiatus, during July–October 2017, an outbreak of the disease occurred in the Peloponnese region in southern Greece that resulted in 48 laboratory-confirmed cases and 5 human deaths (*2*). In 2018, cases further expanded, with a total of 243 human cases and 50 deaths reported from various areas of Greece (*3*).

In June 2017, one month before human cases occurred, dead wild birds were reported in the Argolida regional unit in the Peloponnese region of Greece. Through mid-July, local residents noticed a reduction of the native wild bird population, especially Eurasian magpies (*Pica pica*), hooded crows (*Corvus cornix*), sparrows (*Passer domesticus*), and Eurasian collared doves (*Streptopelia decaocto*). Our team verified the presence of Eurasian magpies with neurologic signs in the area; affected birds were lethargic and unable to fly, stayed low to the ground, and had no reaction to external stimuli (i.e., human presence).

During July and August 2017, we collected a total of 29 dead Eurasian magpies in the study area (Appendix Figure), as part of a monitoring program conducted and supported by the local prefecture since 2016. Twelve of the carcasses were in a condition appropriate for laboratory investigation. 

We extracted brain tissue samples during necropsy for inoculation in Vero cell culture. We vortexed brain homogenates in phosphate-buffered saline and centrifuged them at 4,000 × *g* for 10 min at 4°C. We filtrated 1 mL of brain tissue supernatant with 0.22-μm filters, inoculated it in 75 cm^2^ flasks with 80% Vero cell confluence, and incubated it at 37°C with 5% CO_2_ in the appropriate growth medium. We observed the monolayer daily. When we detected cytopathic effect (in 8/12 samples) ≈48 hours after infection, we transferred the flasks to −20°C for 4 hours. After thawing the supernatant and cells, we performed total RNA extraction using the PureLink RNA Mini Kit (Invitrogen, https://www.thermofisher.com).

We amplified the WNV genome by PCR using a set of 14 primer pairs, newly designed or preexisting from related studies targeting overlapping sequences in the WNV genome (Appendix). Amplicons underwent bidirectional sequencing using the fluorescent BigDye Terminator v3.1 Cycle Sequencing Kit (Applied Biosystems, https://www.thermofisher.com), followed by fragment separation with a 3730xl DNA Analyzer (Applied Biosystems). We verified all nucleotide changes from other WNV strains detected in the 8 positive WNV RNA culture extracts by PCR using the corresponding primers on the tissue extracts. We submitted the consensus sequence, obtained by alignment and assembling in MEGA version 7 software (*4*), to GenBank (accession no. MH549209) and named it Argolida-Greece-2017.

Results of BLAST sequencing (https://blast.ncbi.nlm.nih.gov/Blast.cgi) showed that the Argolida-Greece-2017 strain had the highest sequence similarity (99.79%) to the Nea Santa-Greece-2010 strain (*5*) responsible for the largest WNV human disease outbreak since 2010. Phylogenetic analysis confirmed this closer relatedness to the Nea Santa strain than to other strains within the Hungary/04 cluster (Figure). Our findings indicated possible introduction of the Nea Santa strain in the area of southern Greece and the local genetic evolution that took place before reemergence.

**Figure:**
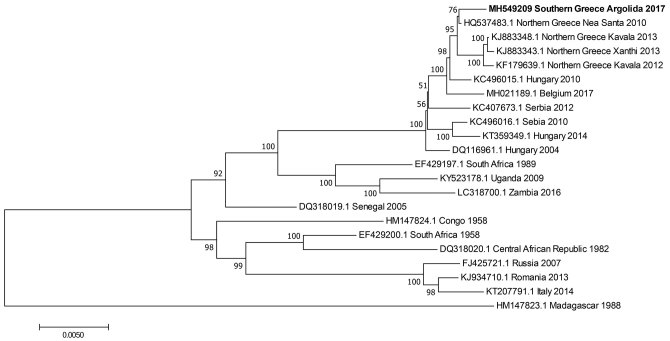
Phylogenetic tree of West Nile virus lineage 2 strains from a Eurasian magpie in Greece (bold) compared with reference strains. Each strain is listed by GenBank accession number, geographic origin, and collection date. Bootstrap values are shown as percentages at each tree node. Scale bar indicates substitutions per site.

The Argolida-Greece-2017 has a total of 23 nt substitutions (3 of them in the 3′ untranslated region of the viral genome) and 4 amino acid changes compared with the phylogenetically closer Nea Santa strain. Amino acid changes include the Ι159Μ in the envelope gene near the NYS glycosylation motif, the H22Y and A298V in the nonstructural (NS) 1 gene, and the K805R mutation in NS5 gene. We predicted that all amino acid changes in the polyprotein gene are tolerated in accordance with the Sorting Tolerant From Intolerant algorithm (*6*). Although these changes do not seem to affect genetic determinants of virulence as was previously reported (*7*), further investigation is needed. The presence of proline at the 249 aa position of the NS3 gene is a mutation related to increased viremia potential and virus transmission rates in corvids (*8*).

In a recent study, Jiménez de Oya et al. performed experimental infection of Eurasian magpies with 2 WNV strains currently circulating in Europe; they found magpies to be highly susceptible to WNV infection, with low survival rates for both strains (*9*). No WNV-associated bird death had been reported in Greece previously, which could be attributed to the lack of an organized wild bird surveillance system in the country. Nevertheless, mass deaths of Eurasian magpies showing neurologic signs, 1 month earlier than a human neuroinvasive outbreak in the area, demonstrate that monitoring sick birds (e.g., using oral swabs or feather pulp) or carcasses of dead wild birds, in an active and passive surveillance system, could benefit public health by recognizing areas in which prevention measures could be implemented to minimize the impact of WNV human disease outbreaks.

Appendix Additional information about bird-virulent West Nile virus, Greece.
