# Supplementary material for: Phylogenetic Analysis of Bird-Virulent West Nile Virus Strain, Greece
Source: Emerg Infect Dis. 2019 Dec;25(12):2323–5. doi: 10.3201/eid2512.181225 (PMC6874256; doi:10.3201/eid2512.181225)
Supplement: Appendix — Additional information about bird-virulent West Nile virus, Greece. [file 18-1225-Techapp-s1.pdf]

Article DOI: https://doi.org/10.3201/eid2512.181225

Phylogenetic Analysis of Bird-Virulent West Nile Virus Strain, Greece

Appendix

Appendix Table. Primers used in analysis of bird-virulent strain of West Nile virus, Greece

Primer name	Sequence primer (5′ →3′)	Nea Santa-Greece-2010 nt position	Bibliography
F1	AGTAGTTCGCCTGTGTGAG	1–19	This study
R1	GCCTTCCAGTACCAGATCAA	1028–1047	
F2	TDGGAATGAGYAACAGRGAC	979–996	(*1*)
R2	TGRTTDATCTGBTGWTCTCCT	2130–2150	
F3	GTTCTCAAGCAATACTGTGAAG	1785–1806	(*2*)
R3	TTCCACCACGACACTCAA	2725–2742	
F4	GGAAGCCATTAAGGATGAG	2673–2691	(*2*)
R4	AAGAACACGACCAGAAGG	3561–3578	
F5	AGAATGGCTGTTGGTATGG	3446–3464	(*2*)
R5	CGTCCTCTCAATCCACAT	4372–4389	
F6	GCAGAACTTGACATAGACTC	4282–4301	(*2*)
R6	TACAGCCGTCCTCAATCT	5257–5274	
F7	ACCTGAAATGTTGAGGAAGA	5151–5170	(*2*)
R7	CGGCATGTTGATGTTGTC	6100–6117	
F8	CATCACCACAGACATATCAGA	5829–5849	(*2*)
R8	AGCCATCCAGCAGAAGAA	6742–6759	
F9	CGGACGCCCTTCAGACAATA	6611–6630	(*2*)
R9	TCGTTACGGCATAGAGCGAC	7044–7059	
F10	CCTGTTGGGACACAAGCCA	6960–6978	This study
R10	CCAGATACAGAAAAGAAGCCATCA	7757–7780	
F11	CAAGAGGGGTGGAGCCAAG	7677–7695	This study
R11	AGGAGAGGCTTCCCTACTGC	8479–8498	
F12	GTGAACATGACAAGCCAGGTG	8380–8400	This study
R12	CTGAGTTCTTCCTACCCAGCC	9179–9199	
F13	GAGTTCGGCAAAGCTAAAGGC	9079–9099	This study
R13	ACACTCTGTTCCAGACTGCG	10128–10147	
F14	TGGATGACGACGGAAGACATG	10105–10125	(*3*)
R14	GGGTCTCCTCTAACCTCTAGT	10825–10845	

References

1. Fall G, Di Paola N, Faye M, Dia M, Freire CCM, Loucoubar C, et al. Biological and phylogenetic characteristics of West African lineages of West Nile virus. PLoS Negl Trop Dis. 2017;11:e0006078. PubMed https://doi.org/10.1371/journal.pntd.0006078

2. Shahhosseini N, Chinikar S, Moosa-Kazemi SH, Sedaghat MM, Kayedi MH, Lühken R, et al. West Nile Virus lineage-2 in Culex specimens from Iran. Trop Med Int Health. 2017;22:1343–9. PubMed https://doi.org/10.1111/tmi.12935

3. Pierre V, Drouet M-T, Deubel V. Identification of mosquito-borne flavivirus sequences using universal primers and reverse transcription/polymerase chain reaction. Res Virol. 1994;145:93–104. PubMed https://doi.org/10.1016/S0923-2516(07)80011-2

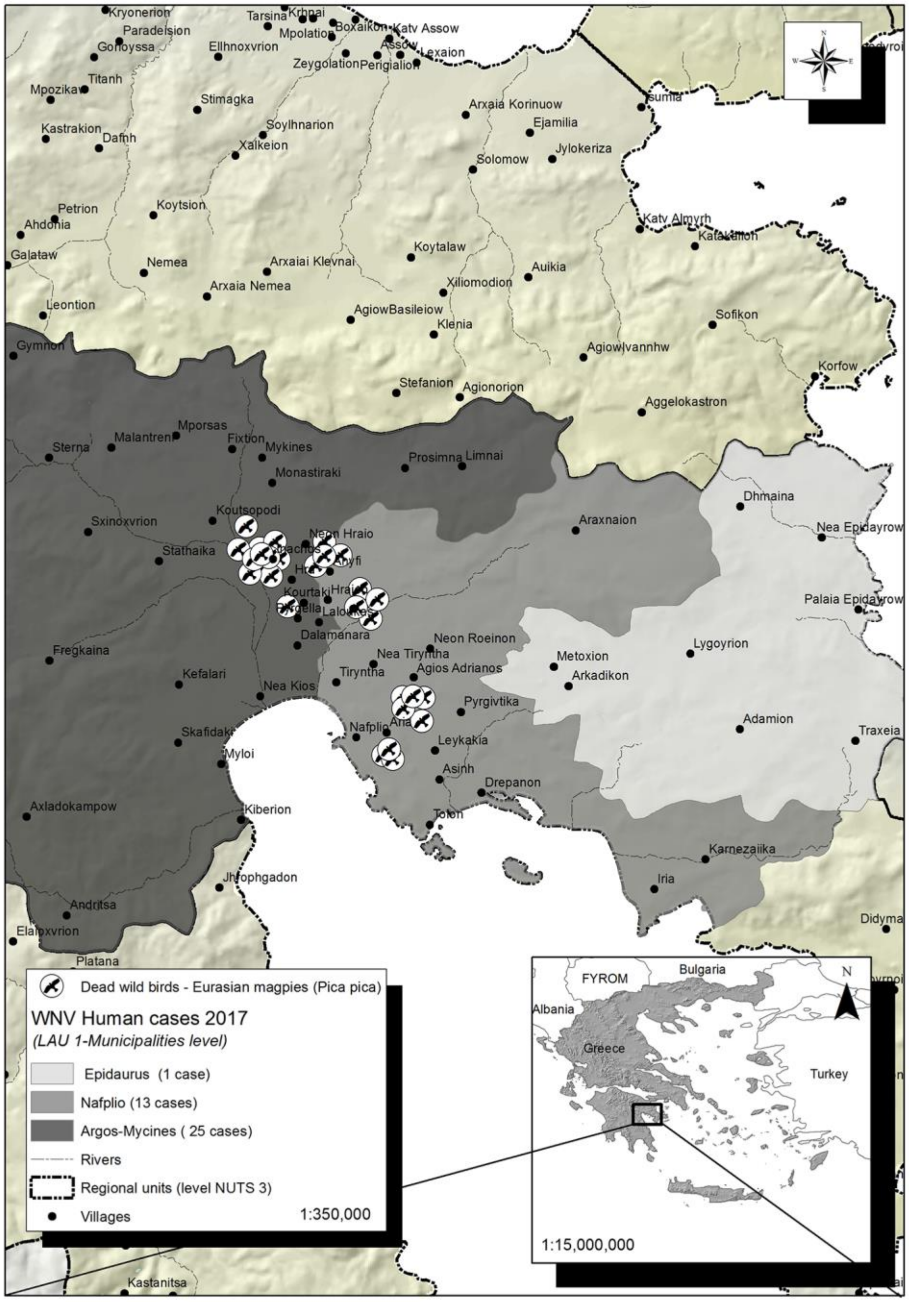

Appendix Figure. Area in which dead Eurasian magpies were collected during July and August 2017 in a study of bird-virulent West Nile virus, Greece.
